# Supplementary material for: How Do Midwives View Their Professional Autonomy, Now and in Future?
Source: Healthcare (Basel). 2023 Jun 19;11(12):1800. doi: 10.3390/healthcare11121800 (PMC10297976; doi:10.3390/healthcare11121800)
Supplement: Supplementary file 1 [file healthcare-11-01800-s001.zip › Document S2 Questionnaire.pdf]

**Supplementary file 2: Questionnaire**
**Dimension: Work content**
I can independently take decisions
I can act independently
I have control over my own work
I get responsibility in the course of my duties
I can do my work according to the professional profile and the relevant legislation
**Dimension: Professionalism of the midwife**
I can take responsibility for my own tasks and decisions
I am responsible and liable for my actions and decisions
I have expertise to act autonomously
I feel competent to act autonomously
I have authority to act autonomously
**Dimension: Relationship with others**
I am not supervised by doctors or other health professionals
I am recognised by society
I am professionally recognised
Other health professionals in maternity care respect the role of the midwife
A legitimately established professional association of midwives defines the rules governing the exercise of their profession. This in consultation with the competent authorities.
**Midwives' views about their autonomy and their views about their autonomy in the future.**
How do you generally rate your autonomy as a midwife?
To what extent do you think that a midwife should be able to work autonomously in Belgium in the nearby future? (regardless of your current work situation)
