# Supplementary material for: How Do Midwives View Their Professional Autonomy, Now and in Future?
Source: Healthcare (Basel). 2023 Jun 19;11(12):1800. doi: 10.3390/healthcare11121800 (PMC10297976; doi:10.3390/healthcare11121800)
Supplement: Supplementary file 1 [file healthcare-11-01800-s001.zip › Document S3 Reliability and construct validity of the autonomy questionnaire.pdf]

Supplementary file 3: Determination of the internal consistency and construct validity of the autonomy questionnaire

Contents

Procedure 1
Visual inspection 2
Reliability 3
Work content 3
Professionalism of the midwife 4
Relationship with others 5
Overarching scale 6
Construct validity 9
Model 1: Model with 3 dimensions 11
Model 2: Hierarchic model 13
Model 3: Single dimension 15
Adaptation basic models 18
Additional analyses 23
Conclusion 23

Procedure

The following analyses were performed in the R programming environment. [1] The visual inspection of the distribution of the items demonstrated that that data were not normally distributed. Secondly, a reliability analysis on each scale's dimension, namely 'work content', 'professionalism of the midwife' and 'relationship with others' was performed using the psych package in R. [2] Cronbach's alpha was used as a measure of internal consistency, as a measure for the reliability of each scale. [3] To consider scale or its dimensions appropriateness, the alpha needed to reach minimal .70, However, .80 is mostly being considered as a good internal consistency rate. The internal consistencies of both 'work content' and 'professionalism of the midwife' were considered good ($\alpha_{workcontent} = 0.80, \alpha_{professionalism} = 0.81$), while the dimension 'relationship to others' reached no acceptable internal consistency rate ($\alpha_{relation\ to\ others} = 0.50$). Dropping an item did not yielded any noteworthy improvement in the alpha coefficient. An item inspection revealed that the items of this subscale are too heterogeneous because of which the scale is not internally consistent.

Next, construct validity of the scale was assessed. Different Confirmatory Factor Analyses (CFA) models were drafted to determine which model fits best the data. [1] As the analyses require structural equation

modelling, data were analysed with the R-package lavaan. [4] The lavaan output contains different fit indices, which allow to evaluate how well the model fits the data. Stated differently, is the model adequate to explain the data? First, if the chi-square test is non-significant, the model fit is considered acceptable since the observed covariance matrix is considered similar to the model-implied covariance matrix. The Comparative Fit Index (CFI) is suitable for small (n < 100) sample sizes (Bentler, 1989). It is advised that the CFI exceeds .90 or, ideally, .95. Next, a value of the Tucker Lewis Index (TLI) between .90 and .95 is considered as a marginal fit, values exceeding .95 represent a good fit (Kenny, 2014). Concerning the Root Mean-Square Error of Approximation (RMSEA) a value below 0.04 describes a good fit and below 0.08 a moderate fit (Kline, 2010).

Next, it was verified per construct if the standardized factor loadings exceed .50. A Maximum Likelihood (ML) estimator resulted in a suboptimal model fit, which is due to the fact that continuous normally distributed data are needed. However, because a 4-point Likert scale was used and as data were skewed, we opted to use the Diagonally Weighted Least Squares (DWLS) estimator, which resulted in good model fits. The detailed results of these CFA models is included in Appendix 1. In sum, the CFA's including three dimensions consistently revealed too low standardized factor loadings for the relationship with others-dimension, indicating that these items cannot be considered good indicators of this latent scale. When excluding this scale from the model, good model fits and standardized factor loadings were consistently obtained.

Visual inspection

The distribution of the variables is looked at.

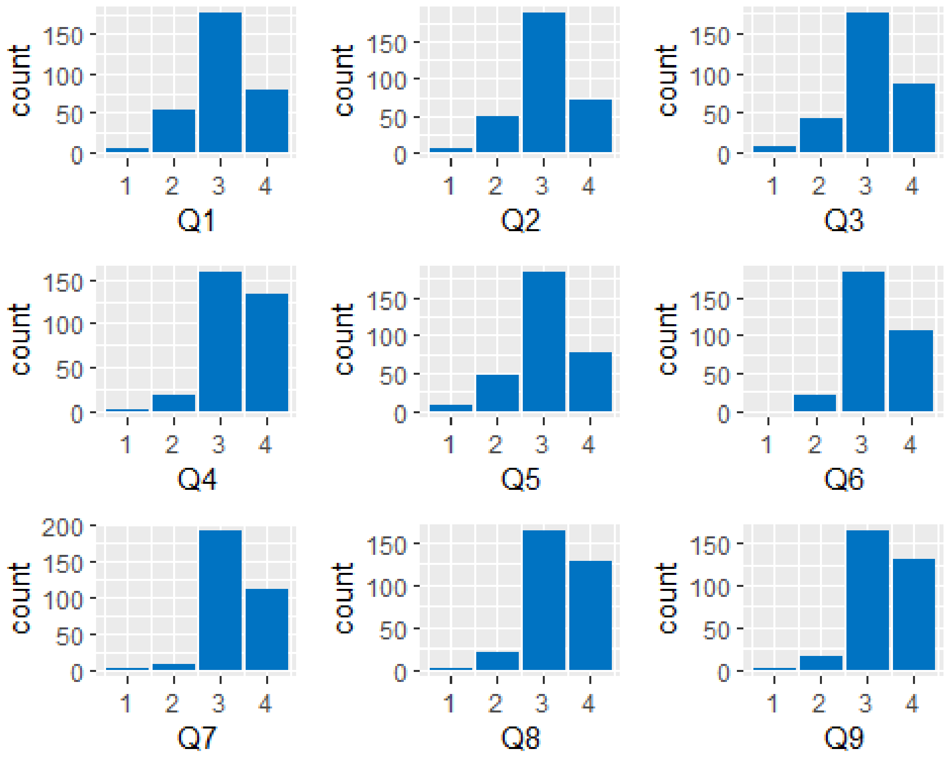

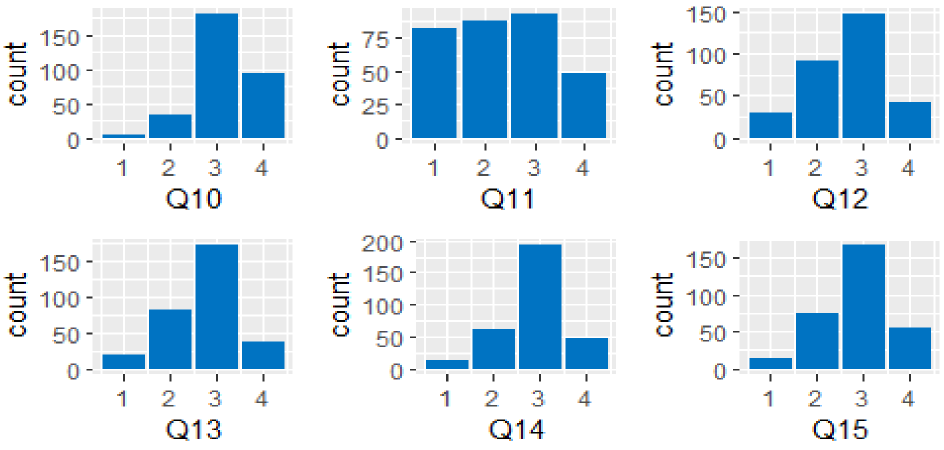

The graphs above show that the data was measured on a 4-point scale and that data are not normally distributed. This is not ideal, this needs to be taken into account.

Reliability

The reliability is then checked on a scale-by-scale basis. Cronbach's alpha is used as a measure for internal consistency. The alpha is ideally above .70 to consider the scale as acceptable. From .80 the internal consistency is considered good.

Work content

```
##
## Reliability analysis
## Call: alpha(x = cbind(rb[, 5], rb[, 6], rb[, 7], rb[, 8], rb[, 9]))
##
## raw_alpha std.alpha G6(smc) average_r S/N        ase mean sd median_r
#          0.8         0.8     0.78        0.45      4 0.018 3.1 0.5        0.44
##
## lower alpha upper            95% confidence boundaries
## 0.76 0.8 0.83
##
## Reliability if an item is dropped:
## raw_alpha std.alpha G6(smc) average_r S/N alpha se var.r med.r
## V1    0.73 0.73 0.68 0.41 2.7  0.025 0.0049 0.43
## V2    0.75 0.75 0.70 0.43 3.0  0.023 0.0060 0.45
## V3    0.78 0.78 0.75 0.47 3.6  0.020 0.0107 0.47
## V4    0.75 0.75 0.71 0.43 3.0  0.023 0.0144 0.41
## V5     0.790.79 0.75 0.49 3.80.019 0.0067 0.46
##
## Item statistics
```

```
## n raw.r std.r r.cor r.drop mean sd
## V1 312 0.81 0.81 0.76 0.67 3.1 0.69
## V2 312 0.77 0.77 0.70 0.62 3.0 0.67
## V3 312 0.71 0.70 0.58 0.52 3.1 0.71
## V4 312 0.76 0.77 0.69 0.62 3.4 0.62
## V5 312 0.68 0.68 0.55 0.48 3.1 0.68
##
## Non missing response frequency for each item
##        1    2    3    4 miss
## [1,] 0.02 0.17 0.56 0.25    0

## [2,] 0.02 0.15 0.61 0.22    0

## [3,] 0.03 0.13 0.56 0.28    0

 ## [4,] 0.01 0.06 0.51 0.43    0

## [5,] 0.02 0.15 0.59 0.25    0
```

A Cronbach's alpha of .80 is observed. This is acceptable, the scale is internally consistent.

Professionalism of the midwife

```
##
## Reliability analysis
## Call: alpha(x = cbind(rb[, 10], rb[, 11], rb[, 12], rb[, 13], rb[,
##     14]))
##
##   raw_alpha std.alpha G6(smc) average_r S/N   ase mean   sd median_r
##        0.81      0.81     0.8      0.45 4.1 0.018 3.3 0.45     0.42
##
## lower alpha upper     95% confidence boundaries
## 0.77 0.81 0.84
## Reliability if an item is dropped:
##      raw_alpha std.alpha G6(smc) average_r S/N alpha se var.r med.r
## V1   0.78   0.78   0.75   0.47 3.5    0.020 0.021 0.48
## V2   0.79   0.79   0.76   0.49 3.8    0.019 0.015 0.49
## V3   0.77   0.77   0.74   0.45 3.3    0.022 0.011 0.42
## V4   0.74   0.74   0.71   0.41 2.8    0.025 0.016 0.36
## V5   0.76   0.76   0.75   0.45 3.2    0.022 0.020 0.42
##
## Item statistics
## n raw.r std.r r.cor r.drop mean sd
## V1 312 0.72 0.73 0.64 0.55 3.3 0.58
## V2 312 0.68 0.70 0.59 0.51 3.3 0.55
## V3 312 0.76 0.75 0.68 0.60 3.3 0.61
```

V4 312 0.82 0.81 0.77 0.69 3.4 0.59
V5 312 0.78 0.76 0.68 0.61 3.2 0.66
##
Non missing response frequency for each item
1 2 3 4 miss
[1,] 0.00 0.06 0.59 0.35 0

[2,] 0.01 0.03 0.61 0.36 0

[3,] 0.00 0.07 0.52 0.41 0

[4,] 0.00 0.05 0.53 0.42 0

[5,] 0.01 0.11 0.58 0.30 0

A Cronbach's alpha of .80 is observed. This is acceptable, the scale is internally consistent.

Relationship with others

```
##
## Reliability analysis
## Call: alpha(x = cbind(rb[, 15], rb[, 16], rb[, 17], rb[, 18], rb[,
##          19]))
##
##        raw_alpha std.alpha G6(smc) average_r S/N   ase mean   sd median_r
##              0.5      0.53    0.53        0.18 1.1 0.046 2.7 0.47     0.09
##
## lower alpha upper      95% confidence boundaries
## 0.41 0.5 0.58 ##
## Reliability if an item is dropped:
##            raw_alpha std.alpha G6(smc) average_r S/N alpha se var.r med.r
## V1    0.59    0.59    0.57    0.27 1.45     0.037 0.043 0.239
## V2    0.33    0.36    0.33    0.12 0.56     0.062 0.018 0.083
## V3    0.32    0.35    0.32    0.12 0.54     0.063 0.014 0.090
## V4    0.41    0.43    0.45    0.16 0.77     0.056 0.043 0.086
## V5   0.51    0.56    0.55    0.24  1.28     0.047 0.053 0.213
##
## Item statistics
## n raw.r std.r r.cor r.drop mean sd
## V1 312 0.51 0.42 0.11 0.084 2.3 1.03
## V2 312 0.69 0.71 0.67 0.425 2.7 0.84
## V3 312 0.69 0.72 0.69 0.459 2.7 0.75
## V4 312 0.58 0.63 0.48 0.333 2.9 0.69
## V5 312 0.45 0.47 0.19 0.146 2.9 0.75
##
## Non missing response frequency for each item
```

```
##      1    2    3    4 miss
## [1,] 0.27 0.28 0.30 0.15    0

## [2,] 0.10 0.29 0.47 0.14    0

## [3,] 0.06 0.27 0.55 0.12    0

## [4,] 0.04 0.20 0.62 0.15    0

## [5,] 0.04 0.24 0.54 0.18    0
```

A Cronbach's alpha of .50 is observed. This is problematic, this scale is not internally consistent. The removal of an item does not lead to a notable increase in the Cronbach's alpha (see reliability if an item is removed).

Overarching scale

Next, it is determined whether the aggregation of all items leads to an overarching reliable scale.

```
##
## Reliability analysis
## Call: alpha(x = cbind(rb[, 5], rb[, 6], rb[, 7], rb[, 8], rb[, 9],
##      rb[, 10], rb[, 11], rb[, 12], rb[, 13], rb[, 14], rb[, 15],
##      rb[, 16], rb[, 17], rb[, 18], rb[, 19]))
##
##   raw_alpha std.alpha G6(smc) average_r S/N   ase mean   sd median_r
##      0.83      0.85    0.88      0.27 5.6 0.014    3 0.39     0.26
##
## lower alpha upper     95% confidence boundaries
## 0.81 0.83 0.86 ##
## Reliability if an item is dropped:
##     raw_alpha std.alpha G6(smc) average_r S/N alpha se var.r med.r
## V1    0.81   0.83   0.86   0.26 4.9     0.015 0.024 0.23
## V2    0.82   0.84   0.86   0.27 5.1     0.015 0.025 0.23
## V3    0.82   0.84   0.87   0.27 5.1     0.015 0.025 0.24
## V4    0.81   0.83   0.86   0.26 4.8     0.015 0.024 0.22
## V5    0.82   0.84   0.87   0.27 5.1     0.015 0.027 0.23
## V6    0.82   0.83   0.86   0.26 4.8     0.015 0.024 0.23
## V7    0.82   0.83   0.86   0.26 5.0     0.015 0.026 0.22
## V8    0.83   0.84   0.87   0.27 5.2     0.015 0.025 0.26
## V9    0.82   0.83   0.86   0.26 5.0     0.015 0.024 0.23
## V10   0.82   0.83   0.86   0.26 4.9     0.015 0.026 0.22
## V11   0.83   0.84   0.87   0.27 5.3     0.014 0.025 0.26
## V12   0.83   0.85   0.87   0.28 5.5     0.014 0.025 0.30
## V13   0.83   0.84   0.87   0.28 5.4     0.014 0.026 0.30
## V14   0.83   0.85   0.88   0.29 5.6     0.014 0.026 0.30
## V15   0.85   0.86   0.88   0.30 6.0     0.013 0.021 0.31
```

```
##
## Item statistics
## n raw.r std.r r.cor r.drop mean sd
## V1 312 0.68 0.68 0.67 0.61 3.1 0.69
## V2 312 0.61 0.61 0.59 0.53 3.0 0.67
## V3 312 0.60 0.59 0.56 0.51 3.1 0.71
## V4 312 0.69 0.71 0.70 0.63 3.4 0.62
## V5 312 0.60 0.60 0.56 0.52 3.1 0.68
## V6 312 0.69 0.71 0.70 0.63 3.3 0.58
## V7 312 0.62 0.64 0.61 0.56 3.3 0.55
## V8 312 0.52 0.55 0.52 0.44 3.3 0.61
## V9 312 0.61 0.64 0.62 0.54 3.4 0.59
## V10 312 0.65 0.67 0.64 0.58 3.2 0.66
## V11 312 0.56 0.52 0.47 0.42 2.3 1.03
## V12 312 0.45 0.43 0.38 0.32 2.7 0.84
## V13 312 0.51 0.50 0.46 0.40 2.7 0.75
## V14 312 0.40 0.40 0.33 0.30 2.9 0.69
## V15 312 0.25 0.23 0.13 0.12 2.9 0.75
##
## Non missing response frequency for each item
##      1    2    3    4 miss ##
[1,]     0.02 0.17 0.56 0.25    0

## [2,]  0.02 0.15 0.61 0.22    0

## [3,]  0.03 0.13 0.56 0.28    0

## [4,] 0.01 0.06 0.51 0.43     0

## [5,] 0.02 0.15 0.59 0.25     0

## [6,] 0.00 0.06 0.59 0.35     0

## [7,] 0.01 0.03 0.61 0.36     0

## [8,] 0.00 0.07 0.52 0.41     0

## [9,] 0.00 0.05 0.53 0.42     0

## [10,] 0.01 0.11 0.58 0.30    0

## [11,] 0.27 0.28 0.30 0.15    0

## [12,] 0.10 0.29 0.47 0.14    0
```

```
## [13,] 0.06 0.27 0.55 0.12      0

## [14,] 0.04 0.20 0.62 0.15      0

## [15,] 0.04 0.24 0.54 0.18      0
```

A Cronbach's alpha of .83 is observed. This is acceptable, this scale is internally consistent.

What about an overarching scale based on only professionalism and work content (i.e. without relationship with others)?

```
##
## Reliability analysis
## Call: alpha(x = cbind(rb[, 5], rb[, 6], rb[, 7], rb[, 8], rb[, 9],
##                 rb[, 10], rb[, 11], rb[, 12], rb[, 13], rb[, 14]))
##
##       raw_alpha std.alpha G6(smc) average_r S/N    ase mean    sd median_r
##         0.86       0.87      0.88          0.39 6.4 0.012 3.2 0.43        0.38
##
## lower alpha upper          95% confidence boundaries
## 0.84 0.86 0.89 ##
## Reliability if an item is dropped:
##             raw_alpha std.alpha G6(smc) average_r S/N alpha se var.r med.r
## V1    0.85     0.85     0.86     0.38 5.6        0.013 0.0136 0.36
## V2    0.85     0.86     0.86     0.40 5.9        0.013 0.0129 0.39
## V3    0.86     0.86     0.87     0.40 6.0        0.012 0.0130 0.38
## V4    0.84     0.85     0.86     0.38 5.5        0.013 0.0137 0.35
## V5    0.86     0.86     0.87     0.40 6.1        0.012 0.0141 0.39
## V6    0.84     0.85     0.86     0.38 5.5        0.013 0.0134 0.36
## V7    0.85     0.85     0.87     0.39 5.8        0.013 0.0142 0.39
## V8    0.86     0.86     0.87     0.41 6.2        0.012 0.0093 0.39
## V9    0.85     0.85     0.86     0.39 5.8        0.013 0.0121 0.38
## V10  0.85     0.85     0.87     0.39 5.8        0.013 0.0141 0.37
##
## Item statistics
## n raw.r std.r r.cor r.drop mean sd
## V1 312 0.73 0.72 0.69 0.64 3.1 0.69
## V2 312 0.66 0.65 0.61 0.56 3.0 0.67
## V3 312 0.64 0.62 0.56 0.52 3.1 0.71
## V4 312 0.75 0.75 0.72 0.67 3.4 0.62
## V5 312 0.62 0.61 0.54 0.50 3.1 0.68
## V6 312 0.74 0.75 0.73          0.67 3.3 0.58
## V7 312 0.65 0.67 0.62          0.57 3.3 0.55
## V8 312 0.58 0.59 0.55          0.48 3.3 0.61
## V9 312 0.67 0.68 0.65          0.58 3.4 0.59
## V10 312 0.68 0.68 0.64          0.59 3.2 0.66
```

```
##
## Non missing response frequency for each item
##       1      2      3      4 miss
## [1,] 0.02 0.17 0.56 0.25    0
## [2,] 0.02 0.15 0.61 0.22    0
## [3,] 0.03 0.13 0.56 0.28    0
## [4,] 0.01 0.06 0.51 0.43    0
## [5,] 0.02 0.15 0.59 0.25    0
## [6,] 0.00 0.06 0.59 0.35    0
## [7,] 0.01 0.03 0.61 0.36    0
## [8,] 0.00 0.07 0.52 0.41    0
## [9,] 0.00 0.05 0.53 0.42    0
## [10,] 0.01 0.11 0.58 0.30   0
```

A slightly increased Cronbach's alpha is observed. This scale is likewise internally consistent.

Construct validity

Then is checked whether there is evidence of the construct validity of the proposed scales. For this purpose, different CFA models are developed to determine which model best fits the data.

Model 1:

- The indicators for the latent variable work content are: Q1, Q2, Q3, Q4 and Q5
- The indicators for the latent variable professionalism are: Q6, Q7,Q8, Q9 and Q10
- The indicators for the latent variable relationship with others are: Q11, Q12,Q13, Q14 and Q15

Model 2 is similar to model 1 but is also a hierarchical structure where work content, professionalism and relationship with others are referred to as indicators of an overarching construct 'autonomy'.

Model 3 is a single factor CFA (i.e., 1 underlying construct is modelled) where all items serve as indicators for the latent construct autonomy (cfr. overarching construct).

```
library(lavaan)
  # Model 1: model with 3 dimensions
  aut <- '
    wc =~ Q1 + Q2 + Q3 + Q4 + Q5
    prof =~ Q6 + Q7 +Q8 + Q9 + Q10
    relationship to others =~ Q11 + Q12 + Q13 + Q14 + Q15
   '
```

```
# Model 2: hierarchic model
hier <- '
  wc =~ Q1 + Q2 + Q3 + Q4 + Q5 prof =~ Q6 +
  Q7 +Q8 + Q9 + Q10 relationship with
  others =~ Q11 + Q12 + Q13 + Q14 + Q15
  aut =~ wc + prof + relationship with others
'


# Model 3: single dimension
one <- '
  aut =~ Q1 + Q2 + Q3 + Q4 + Q5 + Q6 + Q7 +Q8 + Q9 + Q10 + Q11 + Q12 + Q13 +
              Q14 + Q15
'
```

We note that using a Maximum Likelihood (ML) estimator yielded suboptimal results in terms of model fit, this is due to the fact that when using this estimator, continuous normally distributed data are required. However, because only a 4-point Likert scale was used and data were skewed, we opted to use the Diagonally Weighted Least Squares (DWLS) estimator, which resulted in good model fits. The detailed results of these CFA models are included in the additional appendix.

In sum, the CFA's including three dimensions consistently revealed too low standardized factor loadings for the relationship with others-dimension, indicating that these items cannot be considered good indicators of this latent scale. When excluding this scale from the model, good model fits and standardized factor loadings were consistently obtained.

Each of these models is now being evaluated for the model fit. The following conventional rules are used in the evaluation of the model fit. A good model fit meets the following criteria:

- p-value van de chi-square <.05
- CFI > .95
- TLI > .95
- RMSEA < .05
- SRMR < .05

For an acceptable fit, the following criteria apply:

- .90 > CFI > .95
- .90 > TLI > .95
- .05 < RMSEA < .10
- .05 < SRMR < .08

In addition, the standardized factor loads (see below in output the Std.all-column in the Latent Variables section) were also examined if they were more than .50.

Data were analysed using R. [1] As the analyses require structural equation modelling, data were analysed with the R-package lavaan. [4] The lavaan output contains different fit indices, which allow to evaluate how well the proposed model fits the data. First, if the chi-square is not significant, the model fit is considered acceptable since the observed covariance matrix is considered similar to the model-implied covariance matrix. However, this test only seems appropriate for small samples as it will falsely result in a significant result with larger samples. The Comparative Fit Index (CFI) is suitable for small (n < 100) sample sizes. [5] It is advised that the CFI exceeds .90 or, ideally, .95. [6] Next, a value of the Tucker Lewis Index (TLI) between .90 and .95 is considered as a marginal fit, values exceeding .95 represent a good fit. [7] Concerning the Root Mean-Square Error of Approximation (RMSEA) a value below 0.04 describes a good fit and below 0.08 a moderate fit. [8] Next, it is inspected per construct if the standardized factor loadings exceed .60.

Model 1: Model with 3 dimensions

```
# Model 1: model met 3 dimensies

fit_3dimo <- cfa(aut, data = rb, estimator = "DWLS")
    summary(fit_3dimo, fit.measures = T, standardized = T)
```

```
## lavaan 0.6-11 ended normally after 58 iterations ##
##    Estimator                                  DWLS
##    Optimization method                      NLMINB
##    Number of model parameters                   33
##
##      Number of observations                    312
##
## Model Test User Model:
##
##      Test statistic                        216.827
##    Degrees of freedom                           87
##      P-value (Chi-square)                    0.000
##
## Model Test Baseline Model:
##
##      Test statistic                       2697.688
##    Degrees of freedom                          105
```

```
##
##

        P-value                                                    0.000

## User Model versus Baseline Model:
##
##      Comparative Fit Index (CFI)     0.950 ##        Tucker-Lewis
Index (TLI)      0.940
##
## Root Mean Square Error of Approximation:
##
##      RMSEA 0.069
##      90 Percent confidence interval - lower  0.058
##      90 Percent confidence interval - upper  0.081
##      P-value RMSEA <= 0.05                           0.004
##
## Standardized Root Mean Square Residual:
##
##   SRMR                                               0.086
##
## Parameter Estimates:
##
##      Standard errorsStandard
##      Information     Expected
##      Information saturated (h1) model       Unstructured
##
## Latent Variables:
##                           Estimate Std.Err z-value P(>|z|)          Std.lv Std.all
##    wc =~
## Q1  1.000                 0.500                                     0.720
## Q2 0.840 0.061 13.675 0.000 0.420 0.631
## Q3 0.865 0.062 13.926 0.000 0.432 0.606
## Q4 0.948 0.064 14.798 0.000 0.474 0.764
## Q5  0.831                 0.060     13.741    0.000     0.416     0.607
## prof =~
## Q6  1.000                 0.446                                     0.775
## Q7 0.822 0.056 14.761 0.000 0.367 0.662
## Q8 0.723 0.055 13.161 0.000 0.323 0.525
## Q9 0.857 0.060 14.197 0.000 0.382 0.643
## Q10 0.965 0.068 14.302 0.000 0.431 0.656
##relationship to others =~
##      Q11                   1.000                                     0.603     0.584
## Q12 0.485 0.057 8.583 0.000 0.293 0.350
## Q13 0.558 0.058 9.684 0.000 0.337 0.447
## Q14 0.384 0.048 7.989 0.000 0.232 0.333
## Q150.166 0.044 3.811 0.0000.100 0.133
##
## Covariances:

##
```

```
##                                        Estimate Std.Err z-value P(>|z|)              Std.lv Std.all
##      wc ~~
## prof 0.179 0.012 15.202 0.000 0.804 0.804 ## relationship with others 0.237 0.020
11.874 0.000 0.785 0.785
##      prof ~~
##relationship to others        0.191       0.017      11.507       0.000       0.709       0.709
##
## Variances:
                                          Estimate Std.Err z-value P(>|z|)              Std.lv Std.all
.Q1       0.233    0.046    5.091    0.000    0.233    0.482
## .Q2  0.266    0.043    6.214    0.000    0.266    0.601
## .Q3  0.322    0.049    6.600    0.000    0.322    0.633
## .Q4  0.161    0.038    4.174    0.000    0.161    0.417
## .Q5  0.296    0.045    6.651    0.000    0.296    0.632
## .Q6  0.133    0.029    4.585    0.000    0.133    0.400
## .Q7  0.173    0.031    5.487    0.000    0.173    0.562
## .Q8  0.273    0.031    8.750    0.000    0.273    0.724
## .Q9  0.208    0.031    6.619    0.000    0.208    0.587
## .Q10 0.245    0.042    5.819    0.000    0.245    0.569
## .Q11 0.703    0.084    8.421    0.000    0.703    0.659
## .Q12 0.612    0.053    11.638 0.000    0.612    0.877
## .Q13 0.454    0.050    9.000    0.000    0.454    0.800
## .Q14 0.429    0.046    9.414    0.000    0.429    0.889
## .Q15 0.560    0.045    12.570 0.000    0.560    0.982
## wc    0.250    0.025    10.197 0.000    1.000    1.000
## prof  0.199    0.018    11.040 0.000    1.000    1.000
##relationship to others        0.364       0.063       5.822       0.000       1.000       1.000
```

The model fit is on the edge. When inspecting the standardized factor loads, one see that these are far too low for the third dimension (relationship with others). This model is not usable.

Model 2: Hierarchic model

```
fit_hiero <- cfa(hier, data = rb, estimator = "DWLS") summary(fit_hiero,
           fit.measures = T, standardized = T)
```

```
## lavaan 0.6-11 ended normally after 46 iterations ##
##      Estimator                                                    DWLS
##      Optimization method                                        NLMINB
##      Number of model parameters                                     33
##
##        Number of observations                                      312
##
## Model Test User Model:
##
##
```

```
##
##

##        Test statistic                                        216.827
##      Degrees of freedom                                           87
##        P-value (Chi-square)                                    0.000
##
## Model Test Baseline Model:
##
##        Test statistic                                       2697.688
##      Degrees of freedom                                          105
##      P-value                                                   0.000
##
## User Model versus Baseline Model:
##
##         Comparative Fit Index (CFI)                            0.950
          Tucker-Lewis Index (TLI)                                0.940

## Root Mean Square Error of Approximation:
##
##        RMSEA 0.069
##        90 Percent confidence interval - lower  0.058
##        90 Percent confidence interval - upper  0.081
##         P-value RMSEA <= 0.05                                  0.004
##
## Standardized Root Mean Square Residual:
##
##    SRMR                                                        0.086
##
## Parameter Estimates:
##
##        Standard errorsStandard
##        Information       Expected
##        Information saturated (h1) model         Unstructured
##
## Latent Variables:
##                                 Estimate Std.Err z-value P(>|z|)          Std.lv Std.all
##      wc =~
##        Q1                      1.000                                      0.500     0.720
## Q2 0.840 0.061 13.675 0.000 0.420 0.631
## Q3 0.865 0.062 13.926 0.000 0.432 0.606
## Q4 0.948 0.064 14.798 0.000 0.474 0.764
## Q50.831 0.060 13.741 0.0000.416 0.607
##      prof =~
##        Q6                      1.000                                      0.446     0.775
## Q7 0.822 0.056 14.761 0.000 0.367 0.662
## Q8 0.723 0.055 13.161 0.000 0.323 0.525
## Q9 0.857 0.060 14.197 0.000 0.382 0.643
## Q10 0.965 0.068 14.302 0.000 0.431 0.656
##relationship to others =~
##        Q11                     1.000                                      0.603     0.584
```

```
## Q12 0.485 0.057 8.583 0.000 0.293 0.350
## Q13 0.558 0.058 9.684 0.000 0.337 0.447
## Q14 0.384 0.048 7.989 0.000 0.232 0.333
## Q150.166              0.044    3.811    0.000    0.100    0.133
##     aut =~
##       wc              1.000                               0.943    0.943
##       prof            0.806    0.083    9.666    0.000    0.852    0.852
##relationship to others 1.065    0.101   10.539    0.000    0.832    0.832
##
## Variances:
## Estimate Std.Err z-value P(>|z|) Std.lv Std.all
## .Q1 0.233 0.046 5.091 0.000 0.233 0.482
## .Q2 0.266 0.043 6.214 0.000 0.266 0.601
## .Q3 0.322 0.049 6.600 0.000 0.322 0.633
## .Q4 0.161 0.038 4.174 0.000 0.161 0.417
## .Q5 0.296 0.045 6.651 0.000 0.296 0.632
## .Q6 0.133 0.029 4.585 0.000 0.133 0.400
## .Q7 0.173 0.031 5.487 0.000 0.173 0.562
## .Q8 0.273 0.031 8.750 0.000 0.273 0.724
##.Q90.208 0.031 6.619 0.0000. 208 0.587
##.Q10 0.245 0.042 5.819 0.000 0.245 0.569
## .Q11 0.703 0.084 8.421 0.000 0.703 0.659
## .Q12 0.612 0.053 11.638 0.000 0.612 0.877
## .Q13 0.454 0.050 9.000 0.000 0.454 0.800
## .Q14 0.429 0.046 9.414 0.000 0.429 0.889
## .Q15 0.560   0.045   12.570  0.000   0.560   0.982
##       .wc     0.028   0.024   1.167   0.243   0.110   0.110
##       .prof   0.055   0.017   3.271   0.001   0.274   0.274
##       .relationship to others  0.112   0.057   1.965   0.049   0.307   0.307
##       aut             0.222    0.028    7.974    0.000    1.000    1.000
```

# third dimension remains inappropriate

Also in the hierarchical model, the fit is on the edge. Nevertheless, evidence is found of an overarching autonomy construct (see standardized factor loads from autonomy). However, this third dimension results in far too low standardized factor loads. This model is not usable.

Model 3: Single dimension

```
fit_1dimo <- cfa(one, data = rb, estimator = "DWLS") summary(fit_1dimo,
fit.measures =T, standardized = T)
```

```
## lavaan 0.6-11 ended normally after 28 iterations ##
##     Estimator                                   DWLS
##     Optimization method                       NLMINB
##
```

```
##
##

##     Number of model parameters                                  30
##
##       Number of observations                                   312
##
## Model Test User Model:
##
##       Test statistic                                  245.579
##     Degrees of freedom                                           90
##       P-value (Chi-square)                                    0.000
##
## Model Test Baseline Model:
##
##       Test statistic                                2697.688
##     Degrees of freedom                                          105
##     P-value                                                   0.000
##
## User Model versus Baseline Model:
##
##        Comparative Fit Index (CFI)                            0.940
##       Tucker-Lewis Index (TLI)                                0.930
##
## Root Mean Square Error of Approximation:
       RMSEA     0.075 90 Percent confidence interval - lower     0.063
##       90 Percent confidence interval - upper  0.086
##      P-value RMSEA <= 0.05                                    0.000
##
## Standardized Root Mean Square Residual:
##
##    SRMR                                                       0.093
##
## Parameter Estimates:
##
##       Standard errorsStandard
##       Information      Expected
##       Information saturated (h1) model         Unstructured
##
## Latent Variables:
##                                    Estimate Std.Err z-value P(>|z|)              Std.lv Std.all
##     aut =~
##       Q1                         1.000                                       0.468       0.674
## Q2 0.843 0.061 13.708 0.000 0.395 0.594
## Q3 0.872 0.062 14.033 0.000 0.408 0.572
## Q4 0.969 0.065 14.992 0.000 0.454 0.732
## Q5 0.840 0.061 13.855 0.000 0.393 0.574
## Q6 0.895 0.060 14.939 0.000 0.419 0.727
## Q7 0.740 0.052 14.208 0.000 0.346 0.625
## Q8 0.629 0.050 12.629 0.000 0.294 0.479
## Q9 0.742 0.054 13.694 0.000 0.347 0.584
```

Q10 0.857 0.060 14.225 0.000 0.402 0.612
Q11 1.095 0.084 13.038 0.000 0.513 0.496
Q12 0.503 0.057 8.823 0.000 0.236 0.282
Q13 0.589 0.057 10.356 0.000 0.276 0.366
Q14 0.401 0.049 8.156 0.000 0.188 0.270
Q15 0.176 0.047 3.713 0.000 0.082 0.109
##
Variances:
Estimate Std.Err z-value P(>|z|) Std.lv Std.all

.Q1 0.263 0.044 6.007 0.000 0.263 0.545

.Q2 0.286 0.042 6.870 0.000 0.286 0.648

.Q3 0.342 0.048 7.170 0.000 0.342 0.673

.Q4 0.179 0.037 4.808 0.000 0.179 0.465

.Q5 0.314 0.044 7.214 0.000 0.314 0.670

.Q6 0.156 0.027 5.741 0.000 0.156 0.471

.Q7 0.187 0.031 6.089 0.000 0.187 0.609

.Q8 0.291 0.030 9.546 0.000 0.291 0.770

.Q9 0.233 0.030 7.765 0.000 0.233 0.659

.Q10 0.270 0.041 6.579 0.000 0.270 0.626

.Q11 0.805 0.063 12.710 0.000 0.805 0.754

.Q12 0.642 0.050 12.765 0.000 0.642 0.920

.Q13 0.491 0.047 10.443 0.000 0.491 0.866

.Q14 0.448 0.044 10.071 0.000 0.448 0.927

.Q15 0.563 0.044 12.691 0.000 0.563 0.988

aut 0.219 0.021 10.548 0.000 1.000 1.000

##

##
##

Again, the model fit is on the edge. Additionally, the standardized factor loads of Q12 up to and including Q15 are far too low.

Adaptation basic models

Adaptation 1 : Removal of the third dimension

```
# model met 2 dimensies
dim2 <- '
            wc =~ Q1 + Q2 + Q3 + Q4 + Q5
            prof =~ Q6 + Q7 +Q8 + Q9 + Q10
           '
           fit_2dim <- cfa(dim2, data = rb, estimator = "DWLS")
              summary(fit_2dim, fit.measures = T, standardized = T)
```

Model 1

```
## lavaan 0.6-11 ended normally after 28 iterations ##
##     Estimator                                        DWLS
##     Optimization method                            NLMINB
##     Number of model parameters                         21
##
##       Number of observations                          312
##
## Model Test User Model:
##
##       Test statistic                             80.576
##     Degrees of freedom                                 34
##       P-value (Chi-square)                          0.000
##
## Model Test Baseline Model:
##
##       Test statistic                           1956.314
##     Degrees of freedom                                 45
##     P-value                                         0.000
##
## User Model versus Baseline Model:
##
##       Comparative Fit Index (CFI)                   0.976
##       Tucker-Lewis Index (TLI) ##                   0.968
## Root Mean Square Error of Approximation:
##

##    RMSEA                                            0.066
##        90 Percent confidence interval - lower       0.048
##        90 Percent confidence interval - upper       0.085
       P-value RMSEA <= 0.05                            0.072
```

```
## Standardized Root Mean Square Residual:
##
##    SRMR                                            0.077
##
## Parameter Estimates:
##
##       Standard errors Standard
##       Information      Expected
##       Information saturated (h1) model        Unstructured
##
## Latent Variables:
##                        Estimate Std.Err z-value P(>|z|)        Std.lv Std.all
##     wc =~
##  Q1 1.000                      0.501                           0.722
## Q2   0.847   0.067   12.568  0.000   0.425   0.638
## Q3   0.851   0.067   12.778  0.000   0.427   0.598
## Q4   0.961   0.071   13.518  0.000   0.482   0.777
## Q5 0.807                      0.064     12.520     0.000      0.405      0.591
##     prof =~
## Q6  1.000                      0.449                           0.779
## Q7   0.804   0.059   13.692  0.000   0.361   0.650
## Q8   0.733   0.059   12.384  0.000   0.329   0.535
## Q9   0.867   0.065   13.288  0.000   0.389   0.653
## Q10  0.949   0.072   13.252  0.000   0.426   0.648
##
## Covariances:
##                        Estimate Std.Err z-value P(>|z|)        Std.lv Std.all
##     wc ~~
##        prof                    0.180      0.013     14.338     0.000      0.802      0.802
##
## Variances:
## Estimate Std.Err z-value P(>|z|) Std.lv Std.all
## .Q1 0.231 0.047 4.944 0.000 0.231 0.479
## .Q2 0.262 0.044 6.004 0.000 0.262 0.592
## .Q3 0.327 0.049 6.642 0.000 0.327 0.642
## .Q4 0.153 0.040 3.858 0.000 0.153 0.397
## .Q5 0.305 0.045 6.811 0.000 0.305 0.651
## .Q6 0.131 0.030 4.392 0.000 0.131 0.393
## .Q7 0.177 0.032 5.595 0.000 0.177 0.577
## .Q8 0.269 0.032 8.496 0.000 0.269 0.713
## .Q9 0.203 0.032 6.333 0.000 0.203 0.573
## .Q10 0.250 0.043 5.874 0.000 0.250 0.580
## wc 0.251 0.026 9.522 0.000 1.000 1.000
##        prof                    0.201      0.019     10.454     0.000      1.000      1.000
```

```
        # fit ok
```

The removal of the third dimension (relationship with others) results in an acceptable model fit with acceptable standardised factor loads.

```
# hierarchisch model
        hier2 <- '
          wc =~ Q1 + Q2 + Q3 + Q4 + Q5 prof
          =~ Q6 + Q7 +Q8 + Q9 + Q10 aut =~
          wc + prof
        '
        fit_hier2 <- cfa(hier2, data = rb, estimator = "DWLS") summary(fit_hier2, fit.measures
        = T)
```

Model 2: Hierarchic model without relationship with others

```
## lavaan 0.6-11 ended normally after 27 iterations
##
##    Estimator                                    DWLS
##    Optimization method                        NLMINB
##    Number of model parameters                     22
##
##      Number of observations                      312
##
## Model Test User Model:
##
##      Test statistic                           80.576
##    Degrees of freedom                             33
##      P-value (Chi-square) ##                   0.000
## Model Test Baseline Model:
##

##      Test statistic                         1956.314
##    Degrees of freedom                             45
##    P-value                                     0.000
##
## User Model versus Baseline Model:
##
##      Comparative Fit Index (CFI)               0.975
##      Tucker-Lewis Index (TLI) ##               0.966
## Root Mean Square Error of Approximation:
##

##   RMSEA                                        0.068
##            90 Percent confidence interval - lower  0.049
##            90 Percent confidence interval - upper  0.087
##    P-value RMSEA <= 0.05                        0.056
##
## Standardized Root Mean Square Residual:
##
```

```
##    SRMR                                                        0.077
##
## Parameter Estimates:
##
##      Standard errors                                       Standard
##     Information                                            Expected
       Information saturated (h1) model                    Unstructured
## Latent Variables:
##                                   Estimate Std.Err z-value P(>|z|)
##     wc =~
## Q1  1.000
## Q2   0.847   NA
## Q3   0.851   NA
## Q4   0.961   NA
## Q5 0.807                        NA
##      prof =~
## Q6  1.000
## Q7   0.804   NA
## Q8   0.733   NA
## Q9   0.867   NA
## Q10  0.949   NA
##      aut =~
##        wc                        1.000
##         prof                     0.993           NA
##
## Variances:
##                                   Estimate Std.Err z-value P(>|z|)
## .Q1   0.231   NA
## .Q2   0.262   NA
## .Q3   0.327   NA
## .Q4   0.153   NA
## .Q5   0.305   NA
## .Q6   0.131   NA
## .Q7   0.177   NA
## .Q8   0.269   NA
## .Q9   0.203   NA
## .Q10 0.250   NA ##   .wc      0.070    NA
##        .prof    0.022    NA
##        aut                       0.182           NA
```

This model has a good fit, but comprises too few observations to calculate all the necessary parameters. Therefore, the result cannot be evaluated.

```
# single dimension
one2 <- '
                    one =~ Q1 + Q2 + Q3 + Q4 + Q5 + Q6 + Q7 +Q8 + Q9 + Q10
                  '
                   fit_one2 <- cfa(one2, data = rb, estimator = "DWLS")
                 summary(fit_one2, fit.measures = T, standardized = T)
```

Model 3: Single factor CFA without relationship with others

```
## lavaan 0.6-11 ended normally after 26 iterations
##
##      Estimator                                          DWLS
##      Optimization method                                NLMINB
##      Number of model parameters                         20
##
##      Number of observations                             312
##
## Model Test User Model:
##
##        Test statistic     102.536 ##      Degrees of freedom     35
##        P-value (Chi-square)                              0.000
##
## Model Test Baseline Model:
##
##        Test statistic     1956.314 ##     Degrees of freedom     45
##      P-value                                             0.000
##
## User Model versus Baseline Model:
##
##        Comparative Fit Index (CFI)      0.965 ##        Tucker-Lewis Index (TLI)
0.955
##
## Root Mean Square Error of Approximation:
##
##        RMSEA 0.079
##        90 Percent confidence interval - lower  0.061
##        90 Percent confidence interval - upper  0.097
##      P-value RMSEA <= 0.05                               0.004
##
## Standardized Root Mean Square Residual:
##
##    SRMR                                                  0.089
##
## Parameter Estimates:
##
##        Standard errorsStandard
##        Information      Expected
```

Information saturated (h1) model Unstructured
##
Latent Variables:
Estimate Std.Err z-value P(>|z|) Std.lv Std.all
one =~
Q1 1.000 0.465 0.669
Q2 0.851 0.068 12.593 0.000 0.395 0.594
Q3 0.860 0.067 12.875 0.000 0.400 0.560
Q4 0.991 0.072 13.739 0.000 0.460 0.742
Q5 0.820 0.065 12.637 0.000 0.381 0.556
Q6 0.920 0.067 13.727 0.000 0.427 0.742
Q7 0.743 0.057 13.056 0.000 0.345 0.622
Q8 0.654 0.055 11.818 0.000 0.304 0.495
Q9 0.769 0.060 12.743 0.000 0.357 0.601
Q10 0.863 0.066 13.132 0.000 0.401 0.610
Variances:
Estimate Std.Err z-value P(>|z|) Std.lv Std.all
##.Q1 0.267 0.045 5.991 0.000 0.267 0.553
.Q2 0.286 0.042 6.762 0.000 0.286 0.647
.Q3 0.349 0.048 7.271 0.000 0.349 0.686
.Q4 0.173 0.038 4.530 0.000 0.173 0.450
.Q5 0.324 0.044 7.398 0.000 0.324 0.691
.Q6 0.149 0.028 5.285 0.000 0.149 0.449
.Q7 0.188 0.031 6.060 0.000 0.188 0.613
.Q8 0.285 0.031 9.221 0.000 0.285 0.755
.Q9 0.226 0.031 7.371 0.000 0.226 0.639
.Q10 0.270 0.041 6.523 0.000 0.270 0.627
one0.216 0.022 9.696 0.000 1.000 1.000

When the items of relationship with others are removed in the single factor model a good model fit is found. Also, standardized factor loads are satisfactory.

Additional analyses

Additional analyses were carried out to determine if satisfactory results could be obtained with more complicated CFA’s.
For example, a two-group CFA looked at whether better results were obtained if participants were divided into groups based on language. This turned out not to be the case.
In addition, a subset of the relationship with others items was worked on, but this too did not produce any better results
Finally, a two-group CFA was used to explore whether a better result could be obtained if participants were divided into primary care midwives and midwives working in a hospital, but this neither did produce any better results

Conclusion

Based on this analysis, it can be concluded that the first and second dimensions can be used in their present form. The scales of both work content and professionalism of the midwife are internally consistent, which is a measure of

scale reliability. Regarding the scale of relationship with others, we cannot confirm internal consistency. The items are too heterogeneous which makes the scale not internally consistent and therefore far too low Cronbach's alpha's were found. Conversely, an overarching construct (with all items belonging to 1 single scale namely autonomy) did provide good internal consistency.

Regarding construct validity, in the models with the three dimensions too low standardized factor loads for relationship to others are consequently observed, which means that the proposed items are not good indicators of this latent scale. Good model fits were obtained when we omit the relationship with others items from the model. Even then the standardized factor loads were always > .50.

Alternatively, autonomy can be measured by means of a single factor model (use all items as a measure for autonomy). Again, this model resulted in appropriate results when the items of relationship with others were removed.

1. R Development Core Team 2004. R: A language and environment for statistical computing. R Foundation for Statistical Computing, Vienna.
2. Revelle W. psych: Procedures for personality and psychological research. 2017.
3. Polit D, Beck C. Essentials of nursing research: Appraising evidence for nursing practice: Lippincott Williams & Wilkins; 2009.
4. Rosseel Y. Lavaan: An R package for structural equation modeling and more. Version 0.5–12 (BETA). *Journal of statistical software* 2012; **48**(2): 1-36.
5. Bentler PM. EQS structural equations program manual. Los Angeles: BMDP Statistical Software. *Inc BentlerEQS: Structural Equations Program Manual1989* 1989.
6. Cook WL. A structural equation model of dyadic relationships within the family system. *Journal of Consulting and Clinical Psychology* 1994; **62**(3): 500-9.
7. Kenny DA. Measuring model fit. 2015. http://davidakenny.net/cm/fit.htm.
8. Kline RB. Principles and practice of structural equation modeling, 3rd ed. New York, NY, US: Guilford Press; 2011.
